# Altered serum cytokines in patients with symptomatic disk herniation and depressive symptoms

**DOI:** 10.3389/fnins.2024.1366559

**Published:** 2024-04-05

**Authors:** Joanna Bielewicz, Beata Daniluk, Piotr Kamieniak

**Affiliations:** ^1^Department of Neurology, Medical University of Lublin, Lublin, Poland; ^2^Institute of Psychology, Maria Curie-Skłodowska University, Lublin, Poland; ^3^Department of Neurosurgery, Medical University of Lublin, Lublin, Poland

**Keywords:** cytokines, depressive symptoms, disk herniation, sickness behavior, neuropathic pain

## Abstract

**Purpose:**

An increasing number of studies have indicated the important role of cytokines in the development of depressive disturbances (DD). In medically ill patients, cytokines can provoked sickness behavior, the signs of which resemble DD. This results in alterations in behavior to limit energy expenditure and redirect it to cope with particular diseases. The aim of our study was to investigate the role of pro-inflammatory IL-6, TNF-α, and IL-1β and anti-inflammatory IL-10 and TGF-β in DD observed in patients suffering from pain caused by disk herniation (DH) qualified for surgery.

**Patients and methods:**

The intensity of DD assessed by using Beck Depression Inventory, pain intensity, and functional impairment were evaluated in 70 patients with DH who were qualified for surgery. Pro-inflammatory serum levels of TNF-α, IL-1, IL-6, anti-inflammatory TGF-β, and IL-10 were measured.

**Results:**

Elevated serum levels of TGF-β, IL-10, and IL-6 were found in the group with moderate and severe depressive symptoms (SD) compared with the groups with mild (MD) or no depressive symptoms (ND). TGF-β levels were negatively correlated with pain intensity, as assessed using the Present Pain Intensity scale in SD. Functional impairment measured using the Oswestry Disability Index was the most advanced in SD group.

**Conclusion:**

Results of our study can suggest association between depressive disturbances and anti-inflammatory cytokines TGF-β and IL-10. Functional impairment of SD group is more severe but serum levels of TGF-β and IL-10, which are involved in the healing processes, are increased.

## 1 Introduction

The observation of mutual interactions among the immune, endocrine, and central nervous systems has been an important finding in recent years. The crucial role in trans-systemic communication relies on cytokines, creating a dense network of reciprocal associations. They belong to a heterogeneous group of molecules with pro-and anti-inflammatory properties that regulate the inflammatory responses. Their activity is controlled by different inhibitors at the levels of production, protein processing and maturation, receptor binding, and post-receptor signaling by different inhibitors (Dinarello, 2007). Cytokines influence the secretion and activities of other substances during complex, chain-reaction, and feedback processes to maintain a desired balance, which is required for homeostasis. Their function is pleiotropic and can be as detrimental as beneficial, depending on the particular physiological or pathological phenomena. The explanation of its role in this particular process makes cytokines an interesting, possible therapeutic target, which promises more specific, personalized treatment (Saxton et al., 2023).

In Smith (1991) announced “The macrophage theory of depression.” Since then, a growing number of studies have indicated an important role for inflammatory cytokines in the development of depression in both medically ill and medically healthy individuals (Felger and Lotrich, 2013). In medically ill patients, cytokines can provoke “sickness behavior,” which resembles depression disorders (Hart, 1988; Felger and Lotrich, 2013). In acute states, cytokines influence neurotransmitters to adapt and react to stressful situations, such as diseases or injuries. This results in alterations in behavior that limits energy expenditure. Preserved energy is redirected and utilized in processes to cope with diseases or injuries. Such a beneficial effect of stress produced by disease or injury is due to the cooperation of the nervous, endocrine, and immunological systems. The goal of this alliance is to identify a crucial target to be treated as a priority and to maintain homeostasis. Proinflammatory cytokines activate the hypothalamic–pituitary–adrenal axis, leading to the release of cortisol and reciprocally suppressing neuroinflammation (Dunn et al., 2005; Miller et al., 2009).

However, prolonged and chronically elevated levels of inflammatory cytokines and persistent alterations in neurotransmitter systems can lead to neuropsychiatric disorders and depression, which can affect therapeutic responses. Meta-analysis has shown that higher concentrations of pro-inflammatory cytokines, such as IL-6 and TNF-α, are observed in subjects with major depression (Dowlati et al., 2010) but the results of published studies remain divergent (Haapakoski et al., 2015).

The aim of our study was to investigate the role of pro-inflammatory IL-6, TNF-α, and IL-1β and anti-inflammatory IL-10 and TGF-β in depressive disorders observed in patients suffering from pain caused by disk herniation (DH) qualified for surgery. It can provide valuable insight into the involvement of cytokines in the process of depression in situation of pain coexistence.

## 2 Materials and methods

### 2.1 Patients

Seventy patients (32 female and 38 male) with DH and lumbar back pain (LBP) and/or lumbar radicular pain (LRP) who were admitted to the Department of Neurosurgery of the Medical University of Lublin were prospectively enrolled in the study. The patients qualified for microdiscectomy. All patients received written and verbal information regarding the study procedure and signed an informed consent form. In accordance with the binding legislation in this field, the Ethics Committee of the Medical University of Lublin in Poland approved the protocol and details of informal consent (approval number KE-0254/148/2020).

The inclusion criteria for microdiscectomy were: (1) The age of patients between 18 and 80 years, (2) The diagnosis of clinically symptomatic DH with persistent pain refractory to conservative, pharmacological treatment. (3) The confirmation of clinical diagnosis by MRI. Pfirrmann Magnetic Resonance Classification of lumbar disk degeneration was applied. This classification is based on using T-2 weighted MRI graded according to disk degeneration. Patients with grades II, III, and IV are mostly qualified for surgery. However the grade of disk degeneration does not commonly correlate with size of DH and pain intensity (PI). Finally the duration of pain caused by DH and PI are the main indications for surgery. MRI was assessed by the same radiologist and neurosurgeon who qualified for microdiscectomy. The exclusion criteria were: (1) Previous corticosteroid therapy during a period of 3 months preceding surgery, (2) the presence of previous spine surgery or spinal stenosis, (3) co-existence of other medical conditions such as: rheumatoid diseases, diabetes, cancer, psychiatric diseases, recent surgery for another reason than DH, pregnancy, alcohol or drug abuse or other disorders with clinical pain presentation or which can affect cytokines profile. (4) Ongoing therapy with antidepressants.

The patients were aged between 18 and 76 years (*M* = 38.84, *SD* = 11.97). The duration of pain ranged from 1 month to 144 months (*M* = 12.53, *SD* = 22.23). Thirty patients (43%) declared themselves as office workers and the rest as laborers.

### 2.2 Clinical assessment

All patients were assessed separately using the Numerical Rating Scale NRS in the back (NRS-B) and leg (NRS-L), Pain Rating Index (PRI), and Present Pain Intensity (PPI) as part of the Short Form Melzack McGill Pain Questionnaire (SF-MPQ), Oswestry Disability Index (ODI), and Beck’s Depression Inventory (BDI). The subjects were evaluated 1 day before the operation.

NRS: The patient was asked to indicate PI using the scale ratio. An 11-point scale was used, with “0” representing “no pain” and “10” representing the “most severe pain imaginable” at the time of assessment (Bielewicz et al., 2022).

ODI: The patient was asked to assess how his leg and back pain affected nine activities: personal care, lifting, walking, sitting, standing, sleeping, employment/housework, traveling, and social life. The answer to the first question is PI according to the necessity of painkiller intake. Each answer is scored from 0 to 5, and the total score ranges from 0 to 50 (Fairbank and Pynsent 2000).

The SF-MPQ consists of a Pain Rating Index (PRI), Present Pain Intensity (PPI), and VAS scores. For the PRI, the patient was asked to describe the sensory and affective qualities of his experience. The descriptors were rated on an intensity scale of 0: none, 1: mild, 2: moderate, and 3: severe. PRI is the sum of the intensity values of the descriptors that characterize pain. PPI is a six-step scale rating PI from 0 (“no pain”) to 5 (“excruciating pain”). The VAS was assessed on a 100 mm horizontal line. The patient is informed that the left end of the scale represents “no pain” and that the right end represents the “most severe pain imaginable.” The patient was then instructed to mark the PI that was currently being experienced (Melzack, 1987).

BDI (Beck, s Depression Inventory). BDI is the screening scale helping to identify depressive symptoms, assesses their severity and distinguishes them from other psychiatric disturbances. Although BDI questions comply with diagnostic criteria of depression it cannot be the only base to diagnose depression (Beck et al., 1961). Verbal instruction and explanation of aim and technique of BDI was provided by investigator. The patient was asked to answer 21 questions regarding mood during the last 7 days. Each answer is rated on a scale of 0–3. The presence and intensity of depressive disturbances were evaluated using the total scores. The Polish version of BDI was applied which indicates ranges of score as follows: 0–11 – lack of depression, 12 to 19 – mild depression, 20 to 25 moderate depression, 26 and more – severe depression (Parnowski and Jernajczyk, 1977). Based on it, thresholds have been established prospectively.

To avoid suggestions which could have affected the results of self-estimated scale of mood assessment, patients have not been informed about the aim of the study and researchers interests into association of pain and depressive disorders.

### 2.3 Immunological assessment

Sample collections: In the morning, 1 day prior surgery, blood samples were collected from all 70 patients fasting. Subjects did not take any medication on day before blood collection. Five milliliters of venous blood were drawn in the morning and immediately centrifuged at 4000 × g for 15 min. Serum samples were immediately frozen at −80°C until analysis.

A commercial enzyme-linked immunosorbent assay (ELISA) kit of high sensitivity version (Human Quantikine ELISA Kit, R&D Systems) was applied for quantitative determination of human IL-1β, IL-6, TNF-α, IL-10, and TGF-β in plasma samples. Eppendorf tubes of 2 mL were used.

The protocol recommended by the manufacturer is as follows. In addition to the procedure ELISA Reader Victor (PerkinElmer, United States) was used. Cytokines concentrations were expressed in pg./ml. The samples were assayed in duplicates.

### 2.4 Statistical analysis

Statistical analyses were performed using IBM SPSS Statistics software (version 28.0). The results are presented as mean, standard deviation, and minimum and maximum values. For intergroup comparisons, we used the non-parametric ANOVA – Kruskal–Wallis and Mann–Whitney *U* tests for two independent samples. Bonferroni correction was applied in multiple comparison tests. Eta squared was used as measure of the effect size for analysis of variance (ANOVA) model. It is defined as the amount of variation explained by the predictor variable in the total variation for the outcome variable. The *r*-Spearman rank correlation coefficient was used to assess the relationships between variables. The significance level *α* = 0.05 was assumed in the analyses.

## 3 Results

### 3.1 Assessment of the presence and intensity of depressive signs by BDI before surgery

The severity of depressive symptoms in the patients ranged from (0 point) to severe depressive signs (35 points) (*M* = 10.11, *SD* = 6.97). Forty-two patients (60%) had no depressive signs (ND group), 22 (31.4%) had mild depressive signs (MD group), and 6 (8.5%) had moderate or severe depressive signs (SD group). Only one participant in the last group had depressive signs of high intensity. The degree of depressive symptoms did not differ between women (*M* = 10.00, *SD* = 6.35) and men (*M* = 10.21, *SD* = 7.54). This was similar (*z* = −0.12, *p* = 0.906).

The average duration of pain was similar between the ND and MD; 12.4 months (*SD* = 18.97) in the first group and 12.23 (*SD* = 29.68) in the second group. Patients with SD had longer duration of pain with an average of 14.5 months (*SD* = 12.86). The difference in the duration of pain between the groups was not statistically significant (χ^2^ = 2.96, *p* = 0.227).

Because of the wide span of patients age 3 groups were compared. In ND group the average age is 38.74 (*SD* = 12.97), in MD 36.23 (*SD* = 8.6), and in SD group 49.7 years (*SD* = 11.27), but there were no significant differences (χ^2^ = 5.26, *p* = 0.072). There was no significant correlation between intensity of depressive symptoms and age (*r*_s_ = 0.033, *p* = 0.784).

Cytokines and PI scale scores in patients with DH are presented in Table 1.

**Table 1:** Levels of cytokines and scores of pain intensity scales in patients with disk herniation (*n* = 70).

	Minimum	Maximum	Median	IQR	Mean	Standard deviation
IL1	0.06	8.66	0.58	1.05	1.44	1.96
IL 6	0.02	6.96	1.36	1.7	1.75	1.51
IL10	0.20	39.35	8.45	10.87	10.62	8.67
TGF	2976.50	392672.00	37621.4	43557.18	49207.94	56375.15
TNF	0.38	92.91	5.05	6.72	11.04	16.92
NRS B	0	8	3	5	3.20	2.83
NRS L	0	10	5	4	4.89	2.82
ODI	3	41	19	14	21.99	9.28
PRI	0	41	13.5	15	14.14	9.58
PPI	0	4	2	1	2.37	0.995
VAS mm	0	95	61.5	35	54.86	24.43

NRS-B, Numerical Rating Scale – Back; NRS L, Numerical Rating Scale- Leg; PPI, Present Pain Intensity; PRI, Pain Rating Index of McGill Pain Questionnaire (SF -MPQ); ODI, Oswestry Disability Index; IQR, Interquartile range.

### 3.2 Cytokines levels in patient groups with different depressive signs of intensity

IL-6 levels were significantly higher in the SD group than in the MD (*p* = 0.013) and ND (*p* = 0.048) groups. Similarly, significantly higher levels of IL-10 (*p* = 0.012) and TGF-β (*p* = 0.016) were observed in the SD group than those in the MD group. Values of Eta-squared of depressive disturbances were moderate for IL-6, IL-10 and TGF-β. This suggests that variability of IL-6, IL-10 and TGF-β is moderately explained by intensity of depressive disturbances. There were no significant changes in the TNF-α or IL-1β levels (Table 2).

**Table 2:** Levels of cytokines in groups of patients without and with different severity of depressive symptoms – results of comparative analyses by Kruskal–Wallis test.

	No depressive symptoms (*n* = 42) *M (SD)* *Me (IQR)* (a)	Mild depressive symptoms (*n* = 22) *M (SD)* *Me (IQR)* (b)	Moderate/severe depressive symptoms (*n* = 6) *M (SD)* *Me (IQR)* (c)	χ^2^ (p)	Pairwise comparisons	Eta^2^
IL-1	1.34 (1.9) *0.63 (1.07)*	1.86 (2.27) *0.66 (2.51)*	0.56 (0.47) *0.57 (0.68)*	2.18 (0.336)	–	–
IL-6	1.65 (1.27) *1.36 (1.64)*	1.57 (1.68) *0.97 (2.1)*	3.04 (2.01) *2.13 (2.46)*	6.14 (0.046)^*^	a-b (0.294) a-c (0.048)^*^ b-c (0.013)^*^	0.062
IL-10	10.74 (7.81) *8.93 (9.95)*	8.19 (8.43) *5.85 (10.04)*	18.71 (11.55) *16.47 (15.09)*	6.93 (0.031)^*^	a-b (0.098) a-c (0.097) b-c (0.012)^*^	0.074
TGF	55462.69 (67140.71) *40180.55 (46788.38)*	31171.69 (25203.81) *22283.65 (31093.55)*	71557.62 (43204.71) *52246.75 (70460.30)*	6.57 (0.037)^*^	a-b (0.083) a-c (0.137) b-c (0.016)^*^	0.068
TNF	8.13 (9.63) *5.57 (6.29)*	14.87 (23.93) *4.47 (6.31)*	17.35 (24.54) *8 (28.01)*	0.40 (0.818)	-	-

^*^Statistically significant results. M, mean; SD, standard deviation; χ^2^, statistic by Kruskal–Wallis test; Eta^2^, effect size; Me, median; IQR, interquartile range.

### 3.3 Comparison of PI and functionality between groups with different depressive intensity signs

The groups with different degrees of depressive signs did not differ in PI according to NRS-L, NRS-B, VAS, PRI, or PPI. There was a significant difference in the functionality between the groups. ODI was lower in ND (*M* = 19.12, *SD* = 8.69) in comparison with MD (*M* = 25.59, *SD* = 8.39) *z* = −14.62 *p* < 0.01 and with SD (*M* = 28.83, *SD* = 9.54) *z* = −19, 61 *p* < 0.05. The Eta squared value indicates that the patient’s level of disability is significantly explained by the severity of depressive symptoms (Table 3).

**Table 3:** Scores of pain intensity scales in groups of patients without and with different severity of depressive symptoms – results of comparative analyses by Kruskal–Wallis test.

	No depressive symptoms (*n* = 42) *M (SD)* *Me (IQR)* (a)	Mild depressive symptoms (*n* = 22) *M (SD)* *Me (IQR)* (b)	Moderate/severe depressive symptoms (*n* = 6) *M (SD)* *Me (IQR)* (c)	χ^2^ (p)	Pairwise comparisons	Eta^2^
NRS B	3.14 (2.80) *3 (5)*	3.36 (2.97) *3 (7)*	3.0 (3.03) *2 (5)*	0.047 (0.977)	–	–
NRS L	4.62 (2.67) *4.5 (4)*	5.64 (2.99) *6 (4)*	4.0 (3.16) *4 (7)*	2.54 (0.28)	–	–
ODI	19.12 (8.69) *18 (10)*	25.59 (8.39) *23.5 (15)*	28.83 (9.54) *32 (20)*	10.29 (0.006)*	**a-b (0.006)** **a-c (0.027)** b-c (0.594)	0.124
PRI	12.33 (8.13) *12 (15)*	16.32 (9.63) *16.5 (14)*	18.83 (16.14) *13 (32)*	2.11 (0.348)	–	–
PPI	2.21 (1.09) *2 (1)*	2.55 (0.80) *2 (1)*	2.83 (0.75) *3 (1)*	2.63 (0.268)	–	–
VAS mm	51.82 (25.65) *60 (42)*	59.89 (22.06) *64 (21)*	56.80 (26.1) *52 (50)*	0.687 (0.709)	–	–

^*^Statistically significant results in bold. NRS- B, Numerical Rating Scale- Back; NRS- L, Numerical Rating Scale- Leg; PRI, Pain Rating Index; PPI, Present Pain Intensity of the Short Form of McGill Pain Questionnaire (SF -MPQ); ODI, Oswestry Disability Index; M, mean; SD, standard deviation; χ^2^, statistic by Kruskal-Wallis test; Eta^2^, effect size; Me, median; IQR, interquartile range.

### 3.4 Correlations of NRS-L, NRS-B, VAS, PRI, and PPI scores with levels of cytokines

Significant correlation was found only between the PPI score and IL-10 level (*r*_s_ = 0.27, *p* = 0.024). A higher PI assessed using PPI was associated with higher levels of IL-10.

### 3.5 Cytokines levels correlation with ODI, NRS-L, NRS-B, VAS, PRI, and PPI scores in SD

TGF-β levels were strongly and negatively correlated with PPI (*r*_s=_ − 0.926, *p* = 0.008) and ODI (*r*_s_ = −0.829, *p* = 0.042). Higher PPI and ODI scores were associated with lower TGF levels. Additionally, IL-6 serum level were strongly and positively correlated with ODI scores (*r*_s_ = 0.943, *p* = 0.005).

### 3.6 Cytokines levels correlated with ODI, NRS-L, NRS-B, VAS, PRI, and PPI scores in MD

IL-10 levels were moderately correlated with NRS-L (*r*_s_ = 0.453, *p* = 0.034) and PPI (*r*_s_ = 0.522, *p* = 0.013) in patients with MD. Higher PPI scores were associated with higher IL-10 levels.

### 3.7 Cytokines levels correlated with ODI, NRS-L, NRS-B, VAS, PRI, and PPI scores in ND

No significant correlation was observed.

### 3.8 Age correlation with NRS-L, NRS-B, VAS, PRI, PPI, and ODI scores and with levels of cytokines

Age of patients was moderately correlated with ODI scores (*r*_s_ = 0.32, *p* = 0.007). There were no correlations between age and results of scales of pain assessment or cytokines levels.

### 3.9 Comparisons between sexes and NRS-L, NRS-B, VAS, PRI, PPI, and ODI scores and levels of cytokines

Comparisons between males and females showed no significant differences in results of assessment by NRS-L, NRS-B, VAS, PRI, PPI, and ODI. NRS-L *z* = 0.053, *p* = 0.957; NRS-B *z* = −1.07, *p* = 0.285; PRI *z* = −0.761, *p* = 0.446; PPI *z* = −1.55, *p* = 0.120; ODI *z* = 0, *p* = 1.0.

Comparisons between males and females showed no significant differences in cytokines levels IL-1 *z* = −1.31, *p* = 0.191; IL-6 *z* = −0.99, *p* = 0.319; TGF *z* = −0.65, *p* = 0.517; TNF *z* = −0.14, *p* = 0.887. Only level of IL-10 in female group (*M* = 13.07, *SD* = 8.62, *Me* = 12.36) was significantly higher than in males (*M* = 8.56, *SD* = 8.26, *Me* = 6.61) – *z* = −2.59, *p* = 0.009.

## 4 Discussion

The results of our study indicate the possible association of TGF-β, IL-10, and IL-6 with the presence of depressive symptoms in DH patients qualified for surgery. The homogeneity of the evaluated patients group (subjects qualified for surgery) is valuable. However, one should be aware that anxiety caused by the expected surgery can influence the results.

Significantly increased levels of TGF-β, IL-10, and IL-6 in SD compared to MD or ND subjects can suggest the role of cytokines in mood disorders in SD group. We cannot exclude that patients belonging to SD had some innate symptoms presented before the beginning of pain, and an imbalance of cytokines resulted in tendencies for a depressive reaction. A prolonged stressful situation of DH can increase cytokines levels which can manifest as depressive symptoms. Inflammatory markers have been reported to be increased in the blood and CSF of patients with idiopathic major depression; however, there are no particular interleukins deemed to be solely crucial in this phenomenon. Cytokines are pleiotropic molecules, and their mutual relationships modulate inflammatory responses, which can be detrimental but also beneficial, promoting neurogenesis and neuroprotection (Kim et al., 2016).

In the SD group, patients were on average 14 months after the onset of pain symptoms, so, measurements were performed after the acute period. During the acute phase, DH initiates the neuroinflammatory process which results in pro-inflammatory IL-1 and TNF-α increased levels. Anti-inflammatory IL-10 and TGF-β activities is observed latter, as a response maintaining homeostasis. Animals models have showed that during the acute phase, simultaneously, significant alterations and remodeling of the extracellular matrix components occurred. In the late phase, the intervertebral disk returned to the molecule expression profile similar to that of the non-involved tissue, probably due to the extensive remodeling process of the extracellular matrix (de Oliveira et al., 2013). Our group consisted of patients with disk degeneration and persistent pain who did not respond to conservative treatment. This can explain that chronic neuroinflammation is ongoing, and the healing processes are insufficient in this group.

No significant differences in PI were found between the ND, MD, and SD groups. PI did not seem to have influence on the results. We cannot exclude, that the presence of other coexistent factors, such as comorbidities of cardiovascular diseases, obesity, or genetic predisposition, could have affected the outcome. Observed impairment of functionality in the SD measured by the ODI scale can be a consequence of depressive disturbances and comorbidities but should not be affected by PI.

TGF-β family seems to be an interesting cytokines, of which an increase is observed in SD. The TGF-β family comprises a large number of structurally related proteins with multiple biological effects which are contextual. Even the same cell type may show different or opposite responses to the ligand under different biological contexts (Morikawa et al., 2016). TGF-β is involved in numerous physiological processes and diseases. It play essential roles in embryonic development, stem cells, cell fate determination, adult tissue homeostasis, and repair processes (stimulating the role of fibroblasts). TGF-β disturbances are observed in pathological conditions such as cardiovascular diseases, fibrotic disorders and cancer (Goumans and Ten Dijke, 2018). TGF-β signaling is a critical component of skeletal development. In mice with conditional deletion of the TGF-β type II receptor gene, there are many defects in the vertebra and intervertebral disks which are missing or incomplete (Baffi et al., 2004). TGF-β is expressed at high levels in normal cartilage, but is almost absent in osteoarthrosis. However, Schroeder et al. (2013) found increased TGF-β level in degenerative disks but not in the acute state. Its supplementation can enhance cartilage repair and is therefore a potential therapeutic tool. The isoform TGF-β1 may play a key role in the repair of injured annulus fibrosus and the subsequent disk degeneration (Peng et al., 2006). Discectomy procedures can be improved by the isoform TGF-β 3 injection (Illien-Jünger et al., 2014). These beneficial results are limited by the unwanted fibrosis and osteophyte formation (Blaney Davidson et al., 2007). TGF-β signaling promotes the infiltration and proliferation of fibroblasts and inhibits collagenolysis, which can lead to fibrosis (Ren et al., 2023).

TGF-β1 is a relevant mediator of nociception and has protective effects against the development of chronic neuropathic pain (NP) by inhibiting the neuroimmune responses of neurons and glia and promoting the expression of endogenous opioids within the spinal cord (Echeverry et al., 2009). Subcutaneous TGF-β1 infusion prevented pain development after sciatica nerve injury by modulating the endogenous opioid system in an animal model (Lantero et al., 2014).

TGF-β plays an important role in neuronal survival and microglial activation. It potentially exerts effects on neurogenesis, which may in turn play a role in the development of mood disorders such as depression and pathological anxiety (Hiew et al., 2021). Neurogenic mechanisms can respond to stressful stimuli in different ways, such as coping with or adapting to stress. Patients with MDD can have increased plasma levels of TGF-β (Kim et al., 2007, 2016), which are lowered after antidepressant treatment.

The results of the study did not find the significant difference in PI between the SD, MD, and ND groups. However, TGF-β increase serum level in the SD group was correlated with a decrease in PPI score, the global six-step scales of PI. This observation can suggest common or overlapping biochemical pathways for depressive disorders and pain. Some theories have proposed a beneficial effect of depressive disturbances on accompanying somatic diseases (Hart, 1988; Felger and Lotrich, 2013). Affected mood results in apathy, which can preserve energy to cope with the underlying medical problems. Findings of our study can be potentially explained by the beneficial effects of depressive symptoms on pain amelioration, and possibly on healing processes.

IL-10 was the next cytokine with the increased level in the SD group. IL-10 functions are mainly immunosuppressive, as described in bacterial and viral infections, and cancer diseases. Its role is to maintain proper integrity and homeostasis of the tissue epithelial layers. IL-10, secreted by lymphocytes Th2, can limit pro-inflammatory responses by reducing the level of IL-6 (Biffl et al., 1996) and increasing the expression of TGF-β in astrocytes, which attenuates tissue damage caused by inflammation (Sabat et al., 2010; Ouyang et al., 2011) including neuronal injuries (Saraiva et al., 2020). The role of IL-10 can differ and is mostly immunoregulatory depending on the particular processes and engagement of other immunosuppressive or immunostimulatory factors.

IL-10 has been suggested play a role in depressive disorders. However, the exact function and mechanism of action have not been fully elucidated. Numerous studies using animal models have shown that IL-10 deficiency is associated with depression-like behavior, whereas IL-10 overexpression reduces it (Laumet et al., 2018; Saraiva et al., 2020). Yang et al. (2021) found that in male mice with depression, anxiety-like behavior increased the cytokines IL-1β and IL-6 and decreased IL-10. They concluded that enhanced depression and anxiety-like behavior in the examined mice could be related to an imbalance in local excitatory and inhibitory transmission, as well as neuroinflammation in the medial prefrontal cortex and amygdala. This imbalance is associated with increased local inflammation. Moreover, other animal model-based studies have shown that IL-10 promotes learning and memory after learned helplessness (Worthen et al., 2020). Some studies have shown that IL-10 secretion and activity can depend on a particular situation involving different coexisting factors. The results of another animal-based study suggested that recruitment of IL-10 limits neuronal damage after traumatic brain injury but in a sex-dependent manner (Gu et al., 2022).

Studies on different human populations are more variable. Some authors proposed IL-10 as a key cytokine in depression (Roque et al., 2009). Dhabhar et al. (2009) showed a higher IL-6/IL-10 ratio, and the apparent absence of a counterbalancing, immunoregulatory increase in IL-10 in response to elevated IL-6 concentration contributes to the pro-inflammatory status what suggested to be associated with MDD. IL-10 lower level on admission is considered as a predictor of post-stroke depression (Chi et al., 2021).

In contrast, another population-based study showed significantly higher serum IL-6 and IL-10 levels in patients with MDD or bipolar disorder (BD) than in a healthy control group (Wiener et al., 2019). Meyer et al. (2011) reported highly elevated IL-10 levels in a group of patients with depression and cardiovascular risk factors. An increase in IL-10 levels was observed in a group of patients affected by post-COVID depression (Lorkiewicz and Waszkiewicz, 2021).

The same molecular pathway is also involved in the occurrence of NP. Deficiencies in T-lymphocyte function and IL-10 production may underlie the high comorbidity between pain and depression. Although many studies confirm the contribution of IL-10-dependent pathways in pain pathophysiology, their function seems to be dependent on other coexistent factors. A study by Khan et al. (2015), based on an animal model, showed that complete nerve transection increased, while partial nerve injury reduced IL-10 levels in the involved nerve and dorsal root ganglia. Perineural inflammation with minimal nerve damage had no effect on IL-10 levels (Khan et al., 2015). However, the results of these experimental studies are interesting, and IL-10 seems to have promising therapeutic properties. IL-10 can alleviate pain by suppressing inflammation but can also produce antinociception through spinal microglial β-endorphin expression (Wu et al., 2018). In animal models, intrathecal injection of IL-10 alleviated neuropathic pain (Milligan et al., 2006).

The human group observations were divergent. Banimostafavi et al. (2021) described a group of patients with low back pain caused by disk degeneration associated with low level of IL-10. Similar results were reported by Li et al. (2016) in patients with chronic back pain. However, Wang et al. (2016) observed that the median protein level of IL-10 tended to be higher in patients with mild sciatica than in those with severe sciatica and in healthy controls. Palada et al. (2019) found that IL-6, IL-10, TNF-α, and IL-1β levels in CSF were unchanged in patients with DH waiting for surgery. In conclusion, there is insufficient evidence to indicate a firm relationship between inflammation and clinical symptoms of sciatica (Jungen et al., 2019).

Worth noting is that IL-10 promotes healing processes (Ouyang et al., 2011) and influenced adipocytes limiting thermogenesis and energy expenditure (Saraiva et al., 2020) the phenomena which are observed in sickness behaviors.

IL-6 increased level was also seen in SD group. It is secreted by almost every human tissue and cell type, including fibroblasts, astrocytes and microglia cells (Biffl et al., 1996). Question remains whether IL-6 is just a marker of injury severity or plays an active role in the modulation of the post-injury immune response. Mechanical trauma results in a consistent elevation of circulating IL-6 level that is proportional to the degree of tissue injury and is associated with infectious complications. IL-6 has dual functions. First, it exerts pro-inflammatory activity; however, it suppresses inflammation in the feedback reaction. Furthermore, the pro-inflammatory effects of IL-6 include the induction of cell growth and proliferation, whereas its anti-inflammatory actions include enhancing glucocorticoid synthesis and inhibiting the expression of TNFα.

Numerous studies indicates IL-6 elevated levels in patients with MDD (Dowlati et al., 2010; Liu et al., 2012; Haapakoski et al., 2015) and its decreased after anti-depressive treatment (Basterzi et al., 2005).

Degeneration of the intervertebral disk is thought to be mediated by the abnormal production of pro-inflammatory molecules secreted by both the nucleus pulposus and annulus fibrosus (Risbud and Shapiro, 2014). However, Samuelly-Leichtag et al. (2022) found no correlation between pain, which is a sign of lumbar radiculopathy caused by nerve root inflammation and pro-inflammatory cytokines in the blood or disk.

Considering the results of our study, we hypothesize that IL-6 increased level may be involved in the observed depressive disorders that accompany disk degeneration. We cannot exclude the possibility that the elevation of IL-6 serum concentration is just a marker of inflammatory reactions. This explanation is less possible because the increase of IL-6 was not observed in other groups with moderate or no depressive signs. What more, IL-6 serum level does not correlate with the PI what can support the previous observations of Samuelly-Leichtag et al. (2022).

### 4.1 Limitations

A small number of patients presented with moderate to severe depressive symptoms. However, significant differences were observed between groups. More detailed data regarding co-morbidities and medications used by patients are valuable for interpretation.

The cytokinic theory of depression claims that depressive disturbances are associated with serum and intrathecal elevated level of certain cytokines. We have chosen several cytokines which are considered mostly as proinflammatory (IL-6, TNF-α, and IL-1β) and anti-inflammatory (IL-10 and TGF-β) to find which one can be associated with depressive disturbances in our particular group of patients with persistent pain. These cytokines are supposed to be involved into possible pathogenesis of depressive disturbances as a response to stressful situation. We cannot excluded that others cytokines can be engaged in the developing of depressive disturbances. Also, TGF-β and IL-10, which are suggested to play role in the repairing and healing processes can contribute to the presentation of depressive symptoms.

The demographic factors of the examined groups were homogeneous; therefore, they could minimally affect the results. This is a strength of this study because the results were not influenced by many factors. This is also a limitation, because the results cannot be translated directly to other populations and are more divergent.

## 5 Conclusion

The results of our study indicated that patients with moderate or severe depressive symptoms and DH who qualified for surgery had increased levels of TGF-β, IL-10, and IL-6. TGF-β and IL-10 have anti-inflammatory properties and are involved in the healing process that can ameliorate PI. This suggests that in this particular group of patients, depressive symptoms can be treated as sickness behavior and can have an adaptive role in a stressful situation and can have a beneficial effect.

The above results seem to be very interesting but should be treated carefully because of the small number of patients in the SD group. However, statistical significance was observed, which should encourage further research into the relationship between cytokines, depression, and pain.

## Data availability statement

The raw data supporting the conclusions of this article will be made available by the authors, without undue reservation.

## Ethics statement

The studies involving humans were approved by the Ethics Committee of the Medical University of Lublin in Poland. The studies were conducted in accordance with the local legislation and institutional requirements. The participants provided their written informed consent to participate in this study.

## Author contributions

JB: Conceptualization, Data curation, Investigation, Writing – original draft, Writing – review & editing. BD: Data curation, Formal analysis, Methodology, Validation, Writing – review & editing. PK: Funding acquisition, Investigation, Project administration, Writing – review & editing.
